# Annular Pancreas: Insights Into the Diagnosis of a Rare Anatomical Malformation in Pediatric Patients

**DOI:** 10.7759/cureus.85702

**Published:** 2025-06-10

**Authors:** Dimitra Boviatsi, Georgios Zoumpoulis, Eleni Koutrouveli, Marina Vakaki, Aggelos Marantos, Amir Shihada, Alexandros Samolis, Dimosthenis Chrysikos, Dimitrios Filippou, Theodore Troupis

**Affiliations:** 1 Department of Anatomy, School of Medicine, National and Kapodistrian University of Athens, Athens, GRC; 2 Department of Radiology, Children’s Hospital ‘Panagiotis and Aglaia Kyriakou’, Athens, GRC; 3 First Pediatric Surgery Department, Children’s Hospital ‘Panagiotis and Aglaia Kyriakou’, Athens, GRC

**Keywords:** annular pancreas, congenital abnormality, diagnosis, duodenal obstruction, pediatric patients

## Abstract

Annular pancreas is a rare congenital abnormality, detected in both pediatric and adult patients. Normally, the pancreas arises from the fusion of two endodermal buds of the caudal foregut, the dorsal and ventral pancreatic buds, during the first weeks of gestation. However, defects in embryonic development can lead to the formation of an annular pancreas. Being an uncommon clinical entity, a high level of suspicion is crucial for early diagnosis. We present a case of an annular pancreas in a neonate who was initially referred to our department with a prominent diagnosis of esophageal atresia. Preoperative ultrasound assessment indicated duodenal obstruction, raising suspicion of an annular pancreas, which was further confirmed by saline-aided ultrasound examination. Hence, duodenal bypass was performed. This report aims to present the diagnostic approach to this anatomical anomaly in pediatric patients and highlight the role of saline-aided ultrasound examination in approaching an accurate diagnosis, without the adverse effects of contrast medium administration or ionizing radiation.

## Introduction

Annular pancreas is defined as a strip of normal pancreatic tissue that usually encircles the descending duodenum, resulting in partial or complete extrinsic duodenal obstruction [1-3]. This anatomical malformation, with an estimated incidence of approximately one in 12,000-15,000 births, was first named by Ecker in 1862 during an anatomical demonstration of a young male cadaver [4,5]. However, an increasing prevalence of annular pancreas has been recently reported, largely due to its incidental detection in CT or MRI scans and endoscopies [1]. Nevertheless, it constitutes a rare congenital anomaly; thus, the radiologist should be mindful of an annular pancreas in cases of duodenal obstruction. Ultrasound (US) is a first-line imaging modality for pediatric patients with suspected annular pancreas [2]. Saline-aided US is a well-tolerated and readily available examination, with a higher diagnostic performance than upper gastrointestinal (GI) studies [6].

We report a rare case of a newborn with esophageal atresia and annular pancreas. This case report was approved for publication by the Scientific Review Board of Children’s Hospital of Athens “Panagiotis & Aglaia Kyriakou”.

## Case presentation

**Figure 1:**
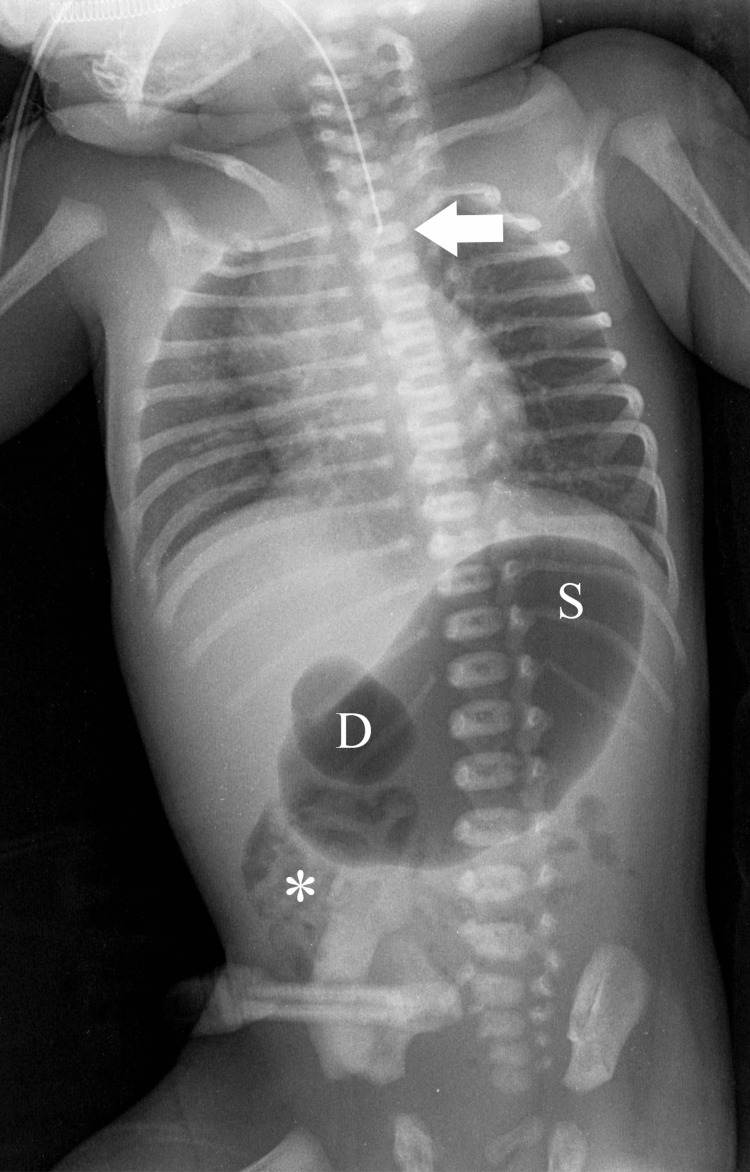
Plain X-ray of the thorax and abdomen indicating esophageal atresia and showing a “double bubble” sign The distal tip of the nasogastric tube (arrow) is superimposed in the midline at the level of the first thoracic vertebra, while distal air in the abdomen (asterisk) is noted, consistent with esophageal atresia with tracheoesophageal fistula. Marked dilation of the stomach and duodenum, namely the “double bubble sign”, is identified. D, Duodenum; S, Stomach

A preterm male neonate (34 weeks and six days of gestation) with a prenatal US diagnosis of a ventricular septal defect (VSD) and polyhydramnios was delivered by cesarean section. Shortly after delivery, the neonate was transferred to our hospital due to excessive drooling and an inability to insert a nasogastric tube beyond 10 cm from the nostril rim, consistent with esophageal atresia. On thorough clinical examination, mild tachypnea with subcostal retractions was noted, without additional abnormal findings. Plain radiography of the thorax and abdomen showed the tip of the nasogastric tube in the proximal esophagus, as well as a “double bubble” sign, necessitating further evaluation of congenital duodenal obstruction (Figure 1).

**Figure 2:**
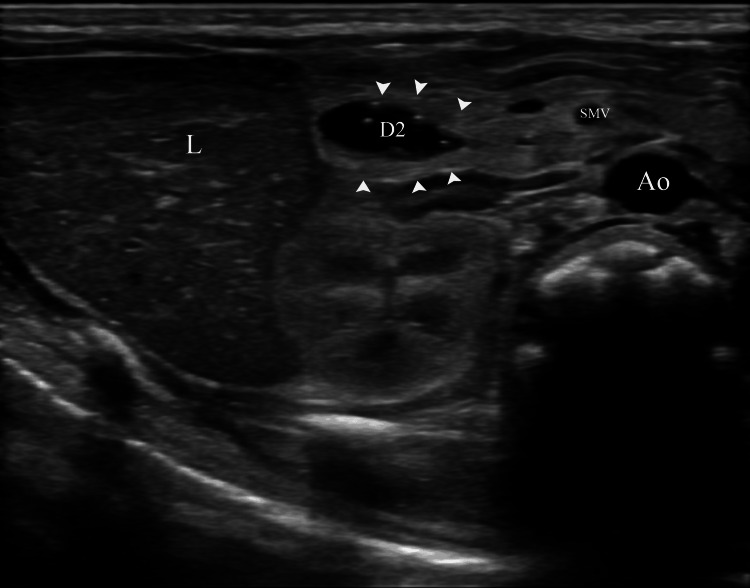
Gray-scale ultrasound image raising suspicion for an annular pancreas Gray-scale transverse plane reveals encircled fluid with strong internal echoes, which is encased by hyperechogenic tissue (arrowheads) of the pancreatic head. Internal echoes account for intraluminal air in the duodenum. Ao, Aorta; D2, Descending duodenum; L, Liver; SMV, Superior mesenteric vein

**Figure 3:**
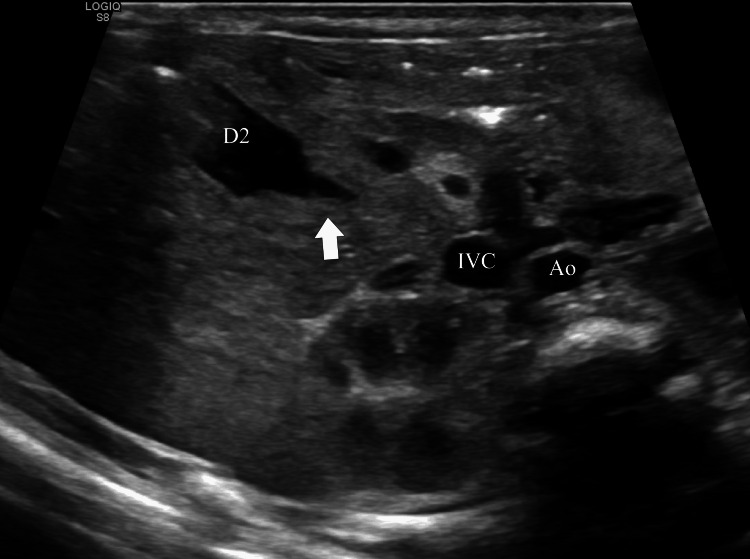
Gray-scale ultrasound plane demonstrating a rim of pancreatic tissue around the duodenum Gray-scale oblique image shows a hyperechogenic band of pancreatic parenchyma encircling the distal portion of the descending duodenum. A beaked appearance of the contour of the distal prestenotic duodenum (arrow) is noted as it enters the pancreatic head. Ao, Aorta; D2, Descending duodenum; IVC, Inferior vena cava

Subsequent US imaging indicated an annular pancreas (Figures 2, 3).

**Figure 4:**
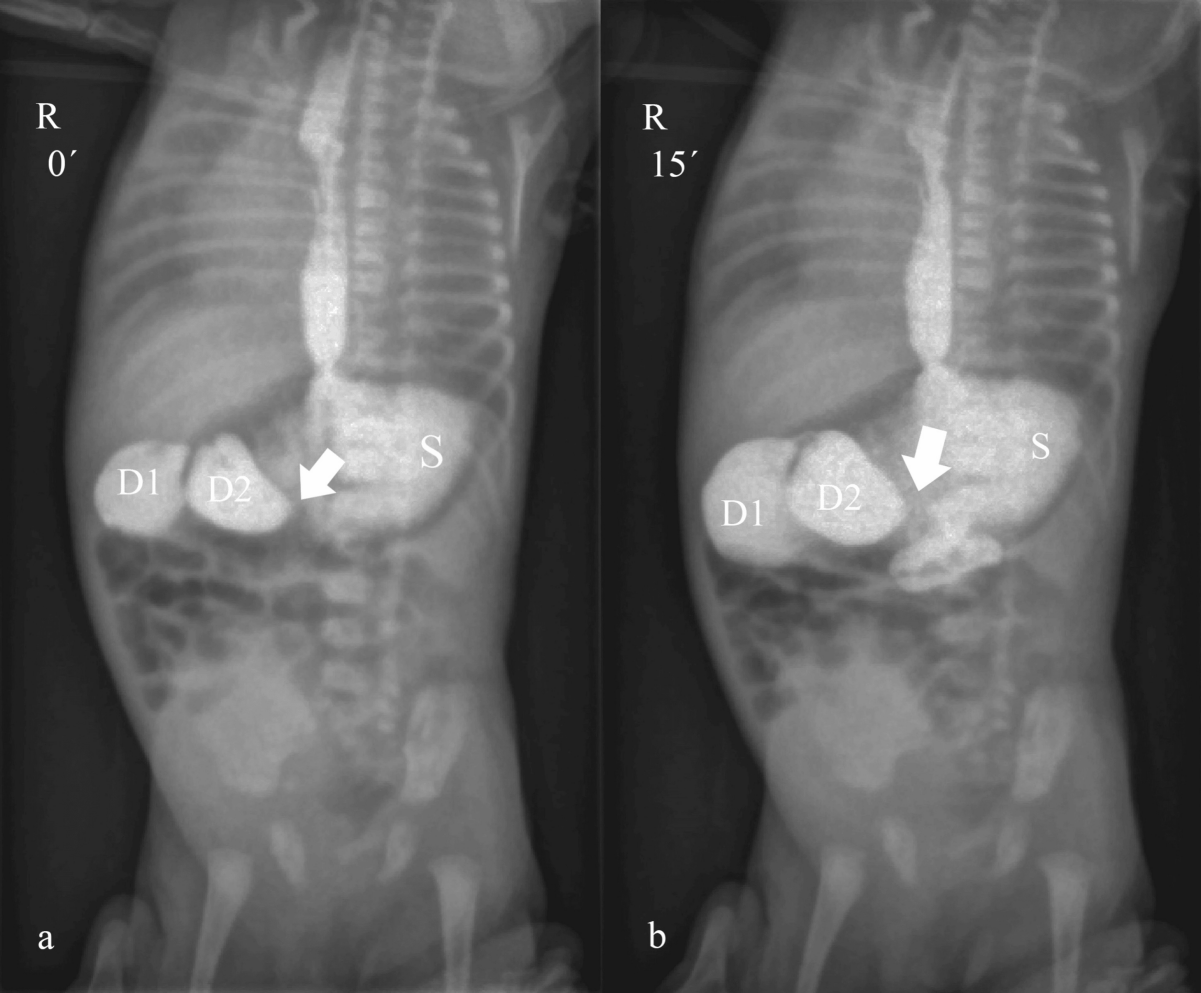
Upper GI study suggesting partial obstruction of the horizontal duodenum (a), (b) The patient is positioned supine on the examining table. (a) Marked dilation of the stomach, duodenal bulb, and descending duodenum is identified, whereas the absence of contrast in the distal duodenum (arrow) is noted. (b) Fifteen minutes after the previous plane, passage of contrast to the distal duodenum and jejunum is seen, apart from a small abruption distally to the dilated descending duodenum (arrow), indicating duodenal stenosis at the horizontal duodenum. D1, Duodenal bulb; D2, Descending duodenum; GI, Gastrointestinal; R, Right; S, Stomach

Preoperative workup also included a transthoracic echocardiogram, which detected an atrial septal defect (ASD) along with a large VSD. The patient was operated on the third day of life and right posterolateral thoracotomy was performed. Type C esophageal atresia was identified, the tracheoesophageal fistula was ligated, and the esophageal continuity was repaired. On postoperative day 12, an upper GI study was conducted, showing marked dilation of the stomach, duodenal bulb, and descending duodenum (Figure 4).

**Figure 5:**
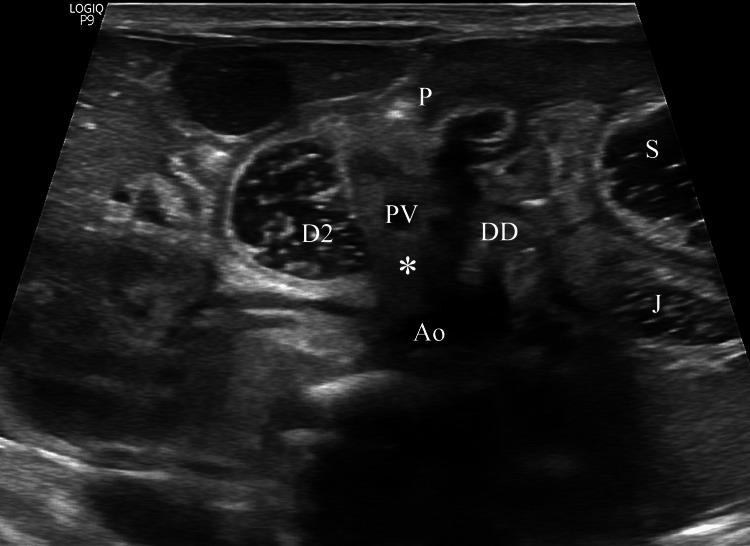
Saline-aided ultrasound image showing an S-shaped formation of dilated and nondilated duodenal segments, along with hyperechogenic tissue at the transition point The gray-scale midline image shows marked dilation of the stomach and descending duodenum with saline, whereas the nondilated distal duodenum crosses the midline anterior to the aorta. The dilated and nondilated segments of the duodenum form an S-shape. A hyperechogenic tissue is recognized (asterisk) anteriorly to the aorta, likely arising from the pancreas and enveloping the horizontal duodenum. Ao, Aorta; D2, Descending duodenum; DD, Distal duodenum; J, Jejunum; PV, Portal vein; P, Pylorus; S, Stomach

**Figure 6:**
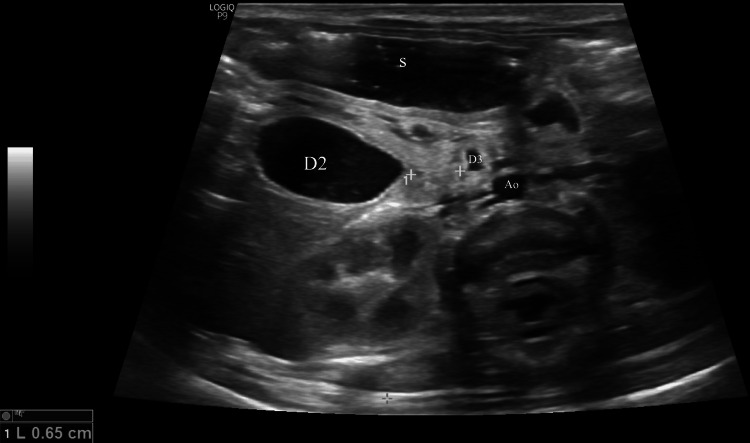
Saline-aided ultrasound image revealing encircled and compressed proximal part of the horizontal duodenum The gray-scale oblique plane demonstrates a dilated prestenotic descending duodenum with a stenotic cone-shaped distal end encircled by pancreatic tissue. The lumen of the horizontal duodenum appears completely obstructed over a 0.65cm segment (between calipers) laterally to the aorta, whereas a normal segment is identified distal to the constricted one, supporting the diagnosis of complete annular pancreas. Ao, Aorta; D2, Descending duodenum; D3, Horizontal duodenum; S, Stomach

A saline-aided US examination was scheduled for postoperative day 14, which revealed normal pancreatic tissue surrounding and compressing the duodenum laterally to the aorta, indicative of an annular pancreas (Figures 5, 6). 

**Figure 7:**
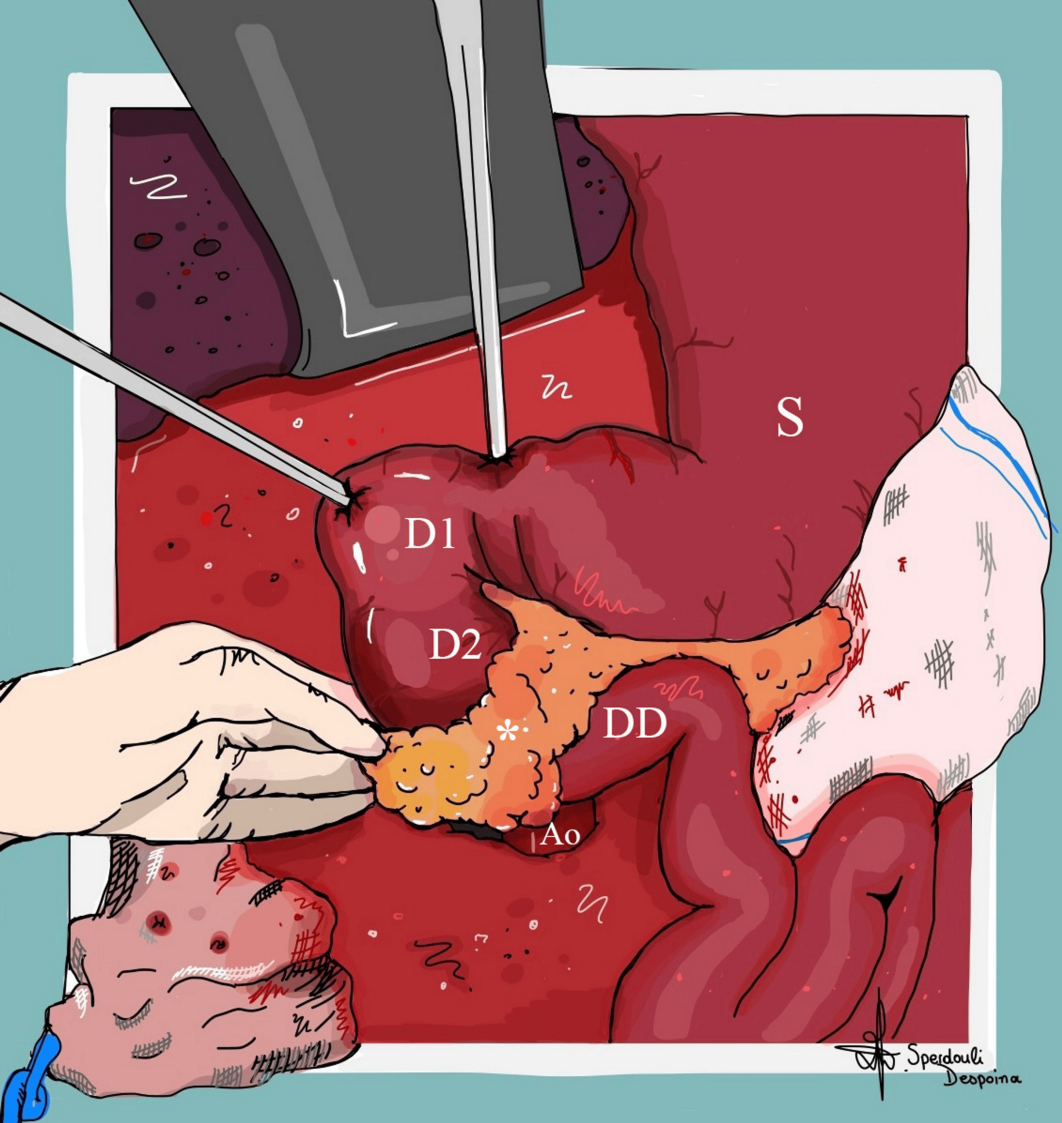
Illustration of the intraoperative view of the annular pancreas in the neonate Dilatation of the stomach, duodenal bulb and descending duodenum is identified, while normal pancreatic tissue (asterisk) encircles the proximal horizontal duodenum, laterally to the aorta. Ao, Aorta; D1, Duodenal bulb; D2, Descending duodenum; DD, Distal duodenum; S, Stomach Image credit: Despoina Sperdouli

The patient underwent laparotomy on postoperative day 18, which confirmed the US diagnosis (Figure 7). 

**Figure 8:**
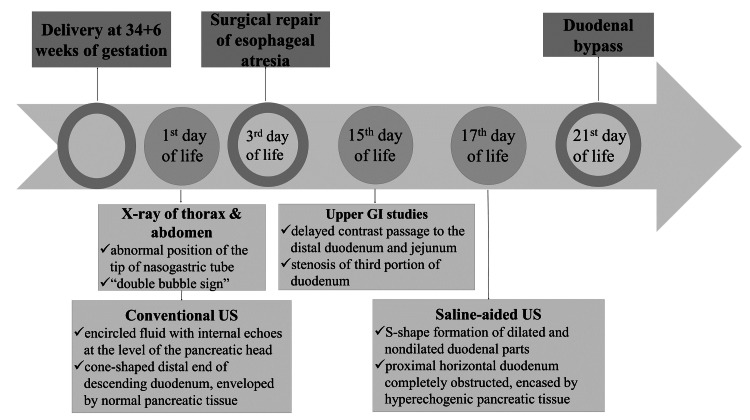
Timeline figure of diagnostic and surgical approach of the neonate, summarizing key diagnostic findings of each modality GI, Gastrointestinal Image credit: Dimitra Boviatsi

Duodenoduodenostomy was performed since it is the surgical treatment of choice for annular pancreas in pediatric patients. The neonate’s intraoperative and postoperative course was uneventful, while the neonate showed no signs of feeding intolerance or significant developmental delay at short-term follow-up. The timeline of the diagnostic approach and surgical management of the patient is presented in Figure 8.

## Discussion

Annular pancreas can partially or completely encase the descending duodenum, affecting neonates, infants, and older children [3,7,8]. Regarding prevalence in male and female patients, a similar ratio was reported in a study by Wang et al., whereas predominant male occurrence was documented in other studies [3,8-10]. 

Although various theories on the embryological origin of annular pancreas have been proposed, the exact pathogenesis remains debatable [1]. The ventral pancreatic bud typically moves dorsally around the duodenum and lies posteriorly and inferiorly to the dorsal bud from the fourth to ninth week of embryonic development [11]. The main hypothesis by Lecco prevails that the ventral pancreatic anlage adheres abnormally to the duodenum, forming an annulus around the descending portion of the duodenum [1]. On the other hand, Baldwin’s theory supports the presence of a bilobed ventral pancreatic bud, thereby attributing the annular formation to a residual left lobe [1]. A modified version of Baldwin’s hypothesis, developed by Kamisawa et al., postulates that the left ventral bud adheres to the duodenum and extends, encircling the descending duodenum [12]. 

Furthermore, annular pancreas is associated with congenital anomalies of the pancreatobiliary tract [13]. As the duodenum rotates to the right and the pancreatic buds develop into the pancreatic parenchyma, both the pancreatic and biliary ducts are formed [11]. The ventral pancreatic bud and primitive common bile duct rotate around the duodenum and lie dorsally to it [11]. Subsequently, the pancreatic buds fuse and the primitive pancreatic ducts integrate with the common bile duct and descending duodenum [11]. Therefore, abnormalities in the pancreas and pancreatobiliary system development can lead to various pancreatobiliary malformations [13]. 

Clinical presentation of annular pancreas has a broad spectrum, depending on the degree of obstruction at birth and presence of coexistent abnormalities, such as duodenal stenosis and atresia [5]. Patients can be asymptomatic, although symptoms can emerge during the neonatal period, infancy, or even later in childhood [1,3]. The dominant symptom is vomiting, which is mostly bilious, whereas non-bilious vomiting is attributed to preampullary duodenal obstruction [5,9,10]. In contrast to adults, pancreatitis is rarely documented in children with annular pancreas, mainly presenting with acute or recurrent abdominal pain related to synchronous pancreatobiliary anomalies [13-15]. Clinical signs of annular pancreas include mild-to-moderate epigastric distension, jaundice, signs of dehydration, weight loss and failure to thrive [5,7,10]. In neonates, it is highly correlated with low birth weight and prematurity [3,5]. Moreover, annular pancreas is commonly associated with concurrent congenital anomalies. The most frequent anomaly is trisomy 21 (Down’s syndrome) [8,15]. Reported congenital abnormalities are listed in Table 1. 

**Table 1:** Classification of congenital anomalies in pediatric patients with annular pancreas Various congenital disorders are frequently coexistent with an annular pancreas, with Down’s syndrome being the most prevalent [1,3,5,8-10,13-15].

Congenital Anomalies	Common	Less Common
Chromosomal abnormalities	Trisomy 21 (mostly)	Trisomy 18
Congenital heart diseases	Atrial septal defect (most common), patent ductus arteriosus, patent foramen ovale	Ventricular septal defect, atrioventricular septal defect, cardiac septal aneurysm, tetralogy of Fallot, and cleft mitral valve
Gastrointestinal anomalies	Intestinal malrotation, duodenal atresia or stenosis or web, esophageal atresia with tracheoesophageal fistula, imperforate anus, Meckel’s diverticulum	Ladd’s bands, intestinal nonrotation, jejunal atresia, ileal atresia, jejunal ectopic gastric mucosa, mobile right colon, situs inversus, omphalocele, and transmesenteric internal hernia
Pancreatobiliary abnormalities	Pancreas divisum	Pancreatobiliary maljunction, choledochocele, and intraluminal duodenal diverticulum
Genitourinary anomalies	Separation of the renal pelvis, dysplastic kidney, ambiguous genitalia	Hydronephrosis, absent left kidney, horseshoe kidney, urethral stenosis, and cryptorchidism
Other	Cardiovascular anomalies and pulmonary artery hypertension	Hydrocephalus, microcephalus, cerebral palsy, spinal cord defect, branchial cleft sinus, agenesis of right lung, asplenia, peripheral vascular anomalies, and polydactyly

A comprehensive diagnostic approach to annular pancreas is pivotal since it can be easily missed in patients with non-bilious vomiting or subtle symptoms [9]. A plain abdominal X-ray is a first-line imaging study, often revealing a “double bubble” sign with or without distal air in the abdomen [9,10]. In the studies of Sencan et al. and Jimenez et al., this characteristic radiological sign is present in 100 % and 88% of cases, respectively [5,9]. Although the classical “double bubble” appearance is pathognomonic for congenital duodenal obstruction, it is non-specific, as its differential diagnosis, apart from annular pancreas, also includes duodenal atresia or stenosis, Ladd bands and volvulus [16]. Moreover, abdominal X-rays can show upper GI obstruction without specific radiological signs [10]. Supplementary upper GI contrast studies can provide helpful information about the level and degree of obstruction [3]. Marked dilation of the stomach, bulb, and descending duodenum with absent or minimal contrast passage in the horizontal duodenum is usually noted [5,10]. 

US comprises a valuable imaging modality for diagnosing annular pancreas antenatally or postnatally due to the lack of radiation exposure [2,17]. Prenatal US demonstrates a “double bubble” sign with or without polyhydramnios, warranting further investigation for congenital duodenal obstruction after birth [3,7,17]. Although a “pliers sign”, an enlarged pancreatic head encasing the duodenum, indicates an annular pancreas, it is less often recognized in prenatal US [17]. On the other hand, the available literature regarding the postnatal sonographic approach to annular pancreas is limited, as it was commonly identified intraoperatively [3,5]. In cases of annular pancreas, conventional transabdominal US mainly reveals a dilated stomach and duodenal bulb, as well as a tubular hyperechoic region in the pancreatic head, which usually pertains to the constricted descending duodenum [2,18]. Encircled fluid, air, or both can be seen in this hyperechoic area [18]. Additionally, a characteristic hyperechogenic band of the pancreatic parenchyma can be detected partially or completely encircling the duodenum [2,17,18]. Nevertheless, assessment for duodenal stenosis or obstruction cannot always be conducted reliably during conventional US examinations, as excessive intraluminal air can distort the imaging [17].

Recent studies by Chen et al. and Yang et al. have demonstrated saline-aided US as a feasible tool for evaluating congenital duodenal obstruction [6,19]. Chen et al. reported higher sensitivity, specificity, and positive predictive value (PPV) of saline-aided US for presence and level of upper GI obstruction than upper GI studies, as well as a statistically significant difference in accuracy for presence of obstruction and in negative predictive value (NPV) for its level (p = .02 and p = .01) [6]. Additionally, Yang et al. estimated sensitivity, specificity, PPV, NPV, and accuracy of the hyperechogenic band for the diagnosis of annular pancreas, using saline-aided US, as 78.8%, 90.3%, 81.2%, 88.8%, and 86.3%, respectively [19]. 

According to Chen et al., two hours of fasting and nasogastric tube placement for decompression are essential before the procedure [6]. In our case, a transanastomotic nasogastric tube was already inserted during the preceding thoracotomy for esophageal atresia. Normal saline was slowly injected through the tube until the stomach was dilated [20]. The exact volume of saline intake is determined by various factors, including vomiting, patient compliance, and adequate lumen distension, as assessed by the radiologist [6]. When performing saline-aided US, alternating patient positioning is crucial to facilitate saline flow from the stomach to the duodenal segments and proximal jejunum [6]. A right oblique position is initially required to observe saline flow from the stomach to the first and second portions of the duodenum [6,20]. Subsequently, saline flow through the third and fourth duodenal segments to the proximal jejunum is monitored in a left oblique position [6]. Furthermore, visualization of the course of the duodenum from the pylorus to the horizontal duodenum across the midline until the duodenojejunal flexure is critical to ensure a comprehensive assessment of the duodenum [21]. When the examination is completed, residual saline is aspirated [6,20]. 

US examination is performed meticulously before and after saline administration to assess the presence and location of duodenal obstruction and to recognize the underlying cause [6,19,20]. In cases of annular pancreas, marked dilation of the pylorus, duodenal bulb with retroperistalsis is mostly noted, compared with the descending, horizontal, and ascending duodenum [19, 20]. Moreover, the pylorus, duodenal bulb, prestenotic, and poststenotic parts of the descending duodenum often form an S-shape [20]. Saline-aided US commonly reveals a hyperechogenic band encircling the constricted duodenum, either partially or entirely [6,20]. Another specific finding is the concave contour of the distal prestenotic duodenum, which often has a beaked appearance as it enters the pancreatic head [6,19,20]. However, the hyperechogenic band can be subtle due to incomplete or thin annular pancreatic tissue, which hinders its detection by US [20]. In such cases, according to Yang et al., the formation of an acute angle between the lateral walls of the prestenotic and poststenotic descending duodenum is a key sonographic finding, with an estimated angle cutoff of 40.7 ° [20]. 

Cross-sectional imaging, including CT and MRI, is rarely used to establish the diagnosis of an annular pancreas in pediatric patients, revealing a rim of pancreatic tissue encasing the descending duodenum [2,14]. On the other hand, ERCP can show an annular duct surrounding the duodenum [13]. MRCP and ERCP are useful for evaluating the structure and course of the pancreatic and biliary ducts to visualize coexisting pancreatobiliary malformations [13,14]. Although advanced imaging studies can detect an annular pancreas in children with duodenal obstruction, administration of contrast media and sedation are required. Additionally, exposure to ionizing radiation during CT and ERCP studies cannot be avoided. 

Definitive treatment of annular pancreas is surgical, aiming to bypass the obstruction while maintaining the continuity of the pancreaticobiliary tract and digestive system [3]. Regarding duodenal bypass, duodenoduodenostomy is the most common surgical technique in pediatric patients since it is associated with fewer complications [3,9]. However, duodenojejunostomy is performed when adequate mobilization of the distal duodenum is not feasible [9].

## Conclusions

Annular pancreas is a rare anatomical malformation that should be considered in the differential diagnosis of congenital duodenal obstruction in neonates and children. Patients present with non-specific symptoms; hence, clinicians and radiologists should be vigilant. Ultrasonography is a well-tolerated and reliable imaging modality for evaluating congenital duodenal obstruction in pediatric patients, without the risks of contrast medium administration and sedation required for advanced imaging studies. Saline-aided US constitutes an inexpensive, easily accessible, and effective imaging approach in such cases, providing real-time information and enabling the radiologist to identify the underlying cause. Given its high diagnostic accuracy, saline-aided US can be used as a first-line preoperative examination in neonates and children with suspected duodenal obstruction, allowing for early detection of congenital anomalies, such as annular pancreas, and optimal treatment planning.
